# Two Rooted Mandibular Second Premolar: An Unusual Finding

**DOI:** 10.7759/cureus.25550

**Published:** 2022-05-31

**Authors:** Akash Sibal, Aditya Patel, Shriya R Singi, Ashutosh Bagde

**Affiliations:** 1 Department of Conservative Dentistry and Endodontics, Sharad Pawar Dental College and Hospital, Datta Meghe Institute of Medical Sciences, Wardha, IND; 2 Research, Jawaharlal Nehru Medical College, Datta Meghe Institute of Medical Sciences, Wardha, IND

**Keywords:** proximal caries, endodontics, root canal therapy, two roots, lower second premolar

## Abstract

Understanding the root and canal anatomy is pivotal before initiating endodontic surgical procedures. Any missed canal will cause treatment failure and ultimately lead to tooth extraction in this era of tooth conservation. Mandibular second premolars have attracted researchers and clinicians for having aberrant anatomy. Variations in the number of roots or canals may not be discerned on 2D radiographs and may become apparent during treatment procedures. The occurrence of two roots in the lower second premolar has been reported in the current case. Here, in this case, the authors have described the clinical course of the patient along with the management of these two rooted mandibular second premolars.

## Introduction

Residual debris after obturating the root canals serves as a nidus for re-infection and subsequent failure of the root canal therapy (RCT). The primary purpose of the endodontic procedures is to eliminate the necrotic debris and microorganisms present in the canals and obturate it with a biologically inert material to achieve a complete three-dimensional seal [1]. The number of canals in a tooth varies based on the number of roots, root dimensions, tooth location, etc. The typical anatomies of all the roots and their canals have been documented in the literature. However, a departure from this typical form may lead to endodontic mishaps. Due to this reason, a thorough knowledge of root morphology is a prime requisite before initiating RCT [2].

The mandibular second premolars have been extensively studied in the literature because of wide-spectrum variations in the root and its canal anatomy. The extra root will possess one or more canals that dental surgeons may neglect. In their systematic review, Wolf et al. reported less than 1.5% occurrence of two rooted mandibular second premolar [3]. The author did an extensive literature review for variations in root numbers about lower second premolars. However, data or a report regarding multiple roots in this tooth was not sufficient. This shows that the dental clinicians might not have frequently encountered additional roots in the lower second premolar. The current study attempts to alert the dental fraternity about such variations in root and canal numbers. It is essential to be aware of such deviations from the normal before initiating RCT.

## Case presentation

A 27-year-old male working as a farmer reported to the department of endodontics at a Rural Tertiary Care Hospital in Central India in August 2021 with a chief complaint of pain and food lodgment in the lower left back region of the jaw for 10-12 days. The general health condition of the patient was good, with no signs and symptoms of systemic diseases. Clinical examination revealed deep distant proximal caries in relation to #35. The patient maintained a good oral hygiene status and did not have any history of deleterious and/or parafunctional habits. The family and past medical history of the patient was insignificant and was not associated with the present complaint. Further, clinically, the patient experienced pain on vertical percussion, which denoted the involvement of the apical region, prognosticating the presence of apical periodontitis. However, no swelling or sinus tract was discernible.

**Figure 1:**
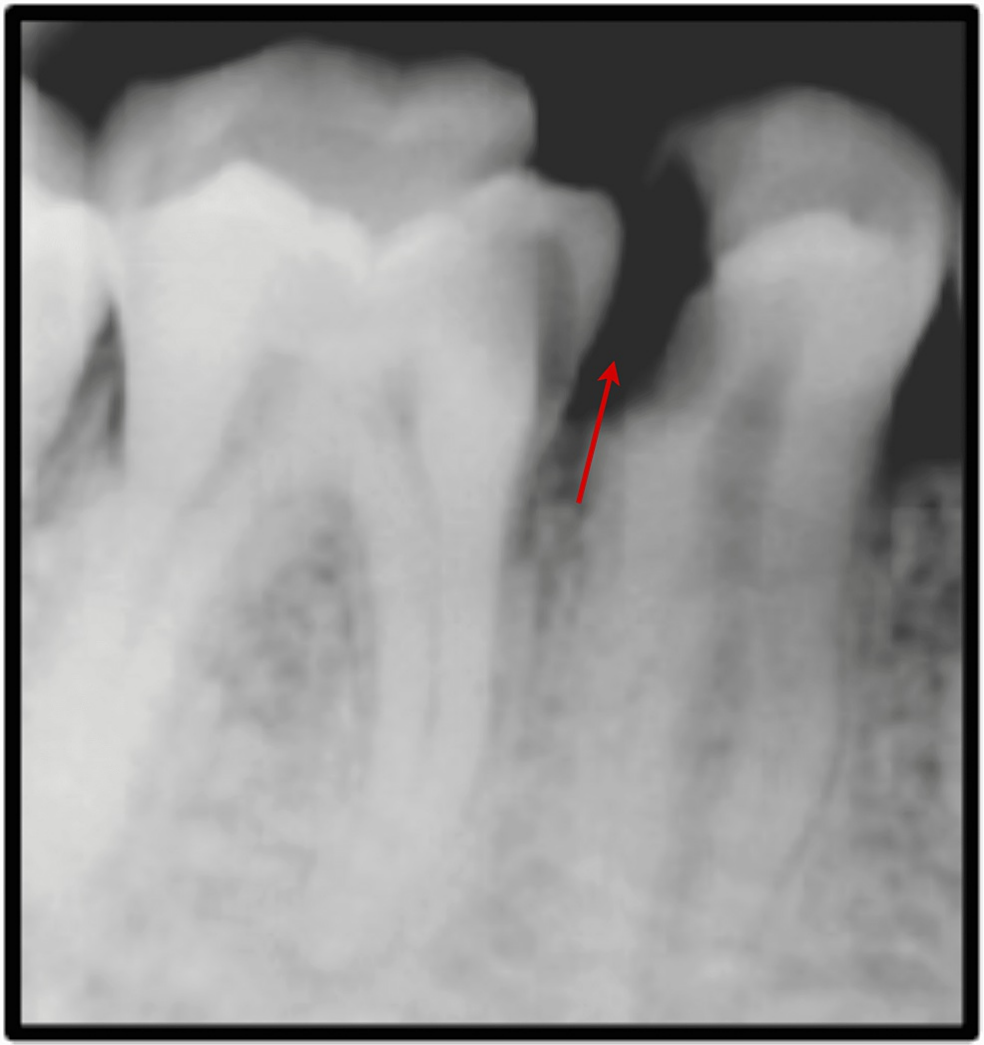
Preoperative view depicting distant proximal caries with #35. Further, an additional root was apparent in this tooth.

An intraoperative periapical radiograph was suggested to the patient to rule out the probability of other diseases like alveolar abscess and symptomatic irreversible pulpitis. On radiographic examination, the authors found two unusual and unexpected findings of the presence of two roots with #35 and no periapical inflammation and abscess. Caries was present on the distant proximal surface of the tooth (Figure 1). A pulp vitality test was also performed, and the pulp showed no response to the electronic pulp tester (Digitest II, Parkell Inc., New Mexico, USA). All these clinical and radiographic analyses confirmed the diagnosis of asymptomatic irreversible pulpitis, indicating removing the necrotic debris from the pulp and restoring the canal space with inert material. As mentioned earlier, two rooted lower second premolars are rare, and the occurrence of an extra root in the present case indicated the need to evaluate the number of canals wisely.

Investigations

1. Vertical Percussion Test

The patient experienced tenderness on percussion, suggesting periapical inflammatory or abscess.

2. Intraoperative Periapical Radiograph

No signs of periapical inflammatory or abscess. The distant proximal wall and partial occlusal wall of #35 were radiolucent because of caries.

3. Pulp Vitality Test

No pulp response to the electronic pulp tester.

**Figure 2:**
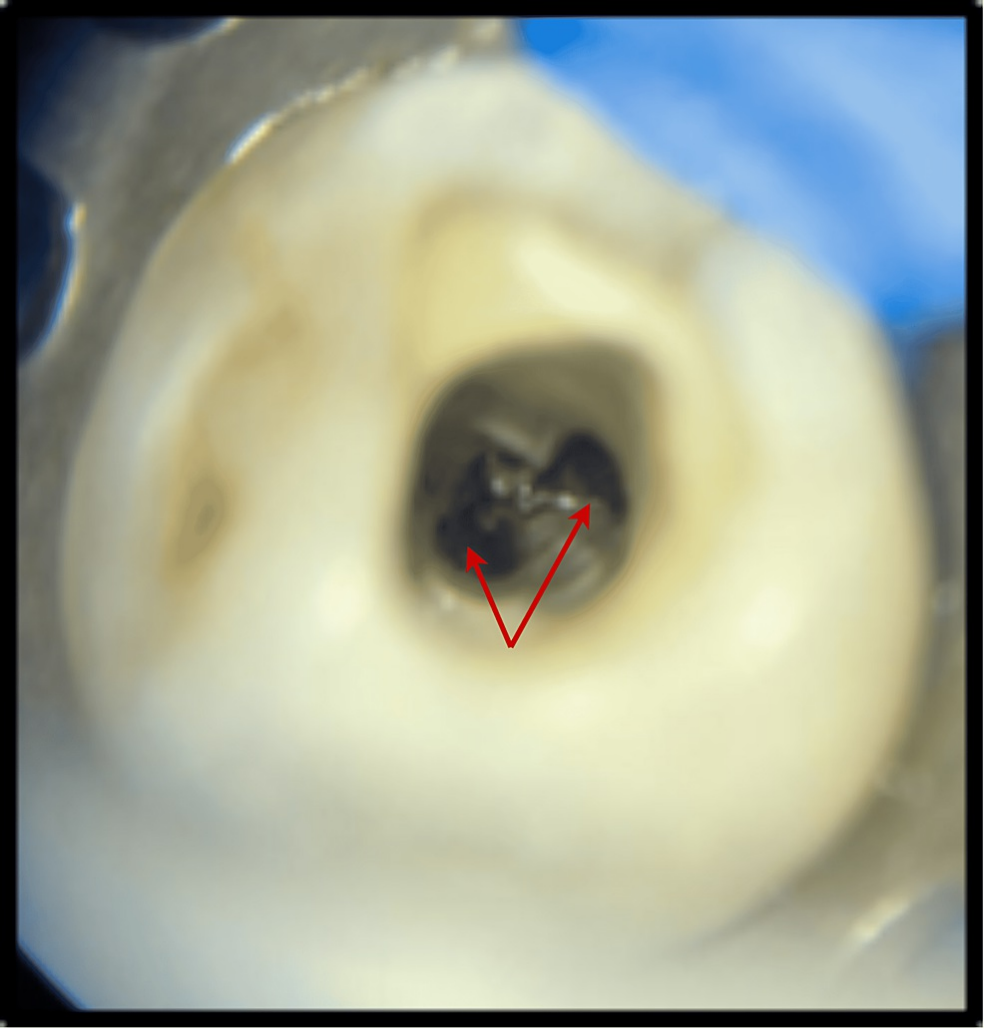
Access cavity preparation, which revealed two canal orifices in separate roots

**Figure 3:**
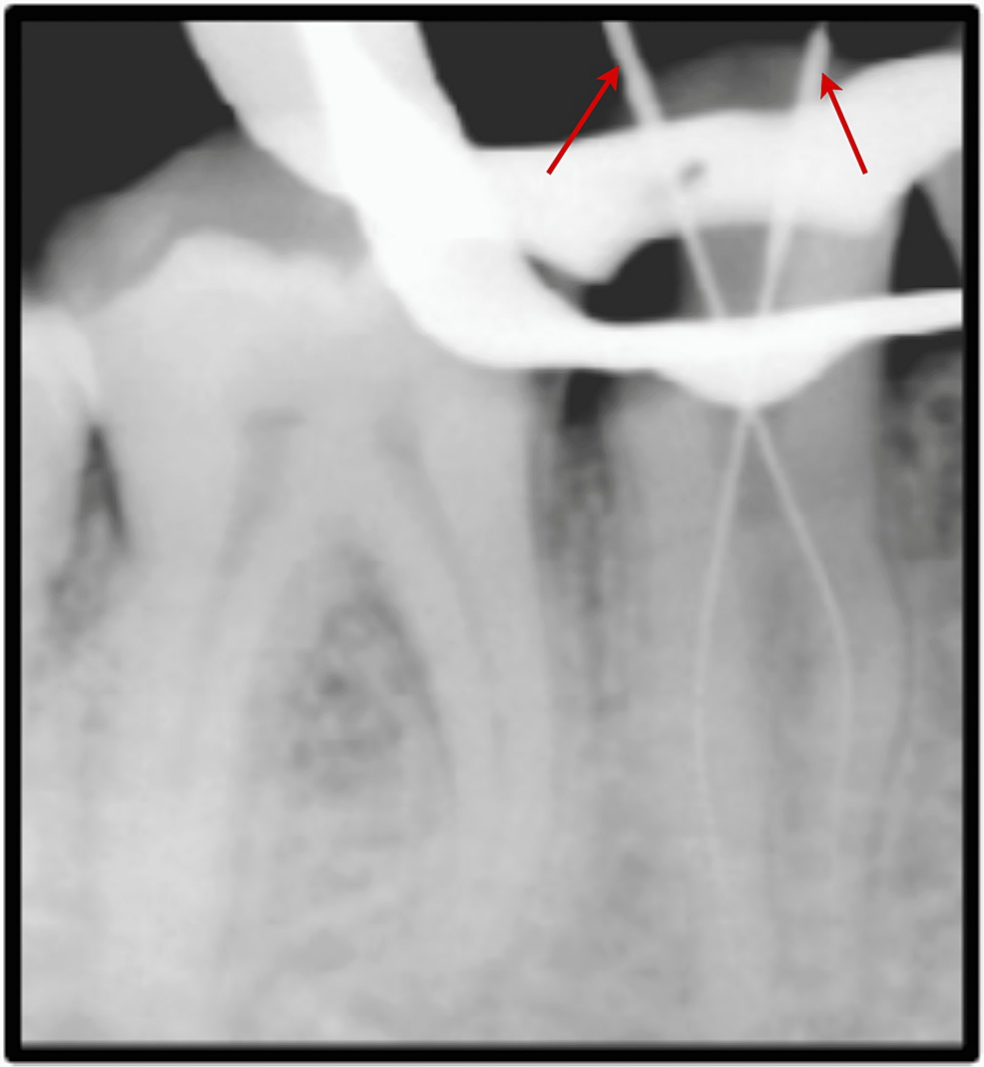
Working length determination

Two roots were found and distinguished as buccal and lingual. Root canal treatment was planned for the patient, and informed consent was obtained before the initiation of treatment. Profound local anesthesia was achieved using lignocaine hydrochloride with adrenaline in the ratio (1:1,00,000). Under proper rubber dam isolation (GDC Fine Crafted Dental Pvt. Ltd., India), complete distant proximal caries was removed, and an access opening was done to expose the pulp canal orifice using 245, 169-L bur (Figure 2). The patency of both the canals in both buccal and lingual roots was confirmed with the #10 K (MANI, Japan) file. Subsequently, the initial working length was determined using #15 K file (MANI, Japan) and Root Zx Mini Apex Locator (J. Morita USA, Inc.) with the help of radiographic images (Figure 3).

**Figure 4:**
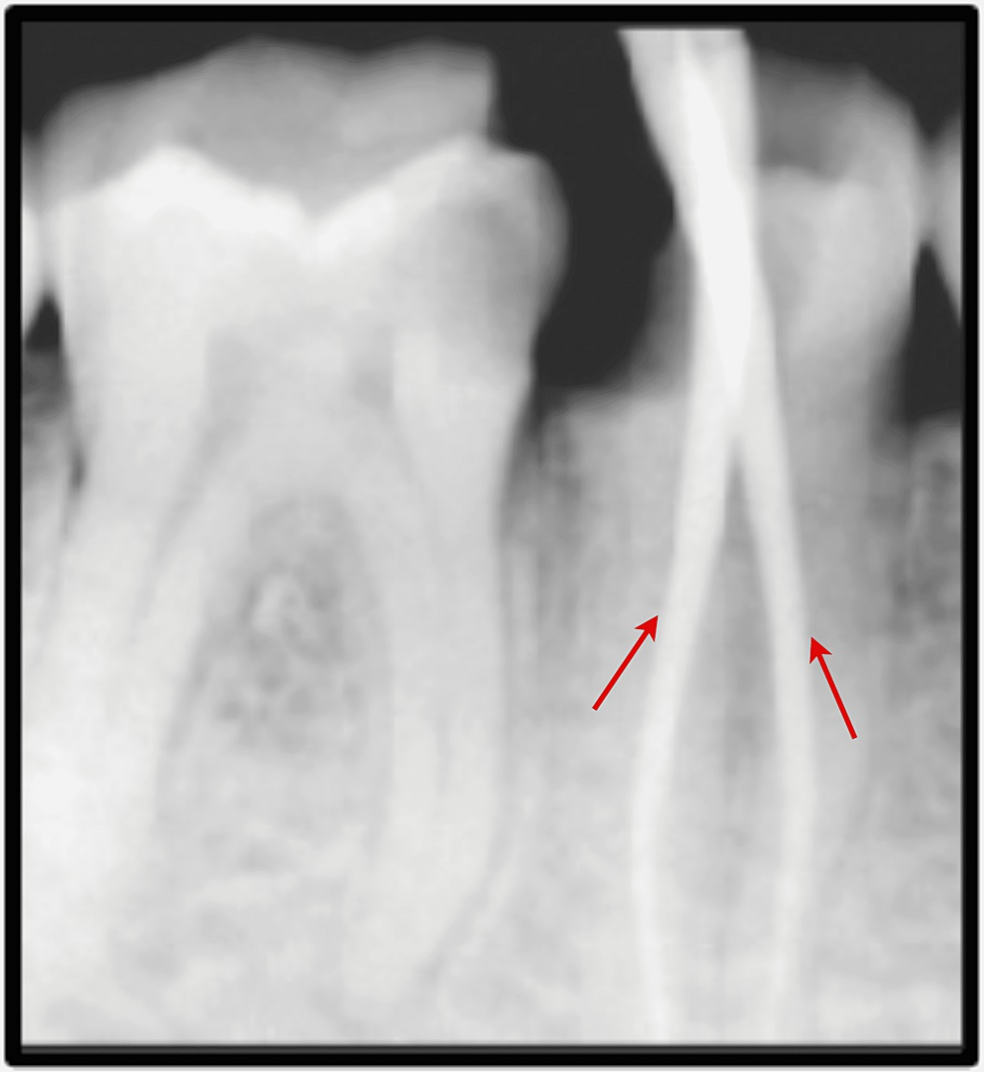
Evaluation of master cone fit

**Figure 5:**
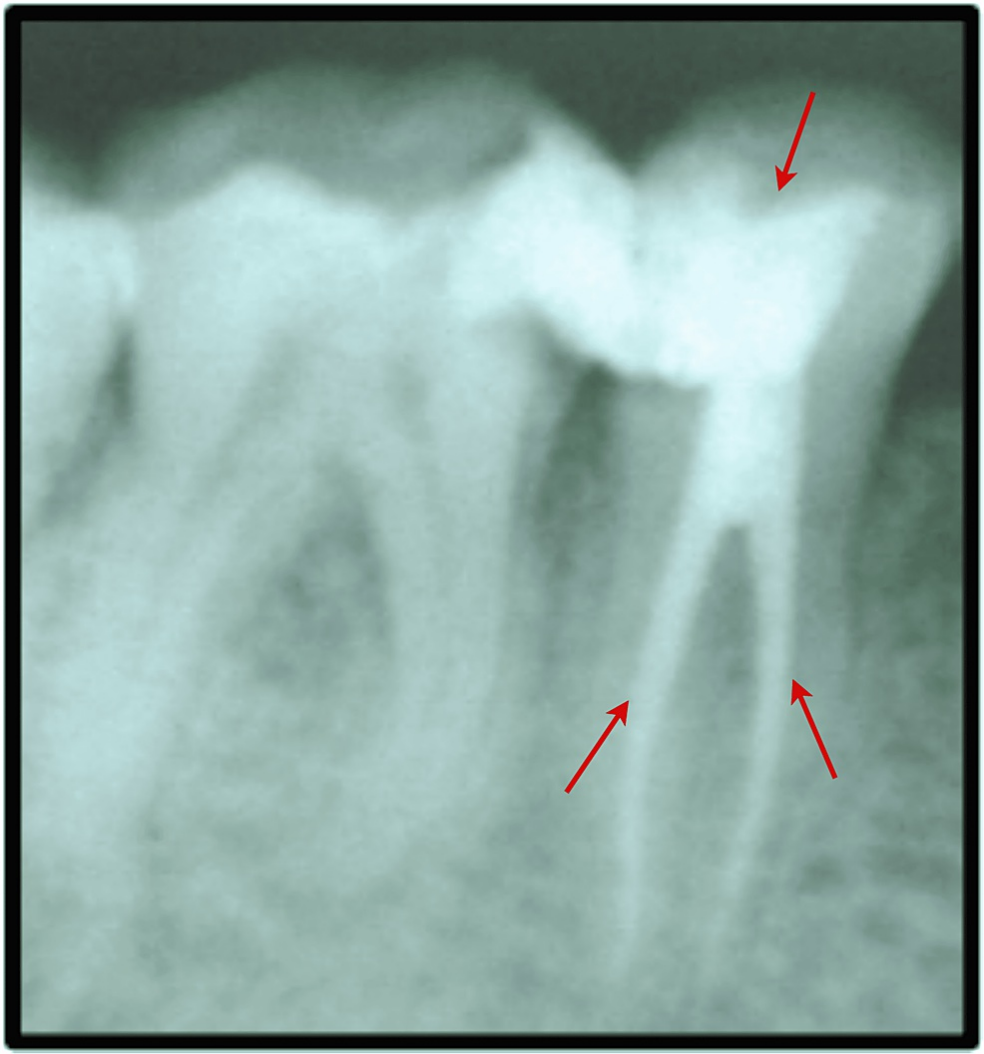
Obturation with an inert material to achieve hermetic seal followed post-endodontic composite restoration

Biomechanical preparation was done using hand-operated files and nickel-titanium rotary instruments (Dentsply, Switzerland). Thorough irrigation with 5.25% NaOCl, normal saline, and 0.2% chlorhexidine was also done simultaneously. The master cone fit was checked and confirmed on the radiograph following the final working length determination (Figure 4). Canals were dried using sterile paper points, and obturation was done with both the roots, followed by post-endodontic composite restoration (Figure 5). A postoperative radiograph was taken, and the complete hermetic seal was verified with a horizontally angulated post-treatment radiograph.

## Discussion

As the complex anatomy of the root and canal systems along with the changes in the internal anatomy of teeth, strategic planning, and aids to diagnose such variable anatomy using preoperative cone beam-computed tomography or multiangle radiographs are very important [4], incidences of an additional root and canals in mandibular premolars may provide a significant endodontic difficulty, thereby increasing the chances of endodontics failures [5,6]. According to research, these findings are clinically critical as the mandibular premolars possess a great failure rate [7]. Failure to detect the existence of additional roots or canals might result in accidents such as acute flare-ups during treatment and endodontic therapy failure. For complete removal of the infection foci and prevention of disease recurrence, negotiation of main canals in all the roots of a tooth is essential [8]. Using various diagnostic aids such as dental microscope and computed tomography techniques may be an effective way to discover missed canals rather than routine radiographs that give a two-dimensional image of the root structure [9,10].

Lower premolars have gained considerable attention from the clinical world due to wide-spectrum occurrences of morphological aberrations in root and canal systems [11]. Not much literature is available regarding the lower second premolar compared with first premolars. Encountering anatomical deviations in second premolars, therefore, may create clinical challenges and symptoms persist for a longer time. Arayasantiparb et al. reported that multiple roots in the first premolar were approximately 5.73%, while surprisingly, the authors did not find any second premolar with additional roots in the Thai population [12]. Dosunmu et al. reported that the prevalence of two rooted mandibular first and second premolars is 1.8%-2.1% and 0.4%, respectively [13]. In this report, it was found that the mandibular second premolar is double rooted.

The current case represents an unusual finding of an additional root with #35 in an adolescent patient. Radiographic interpretation revealed no complex internal anatomy of the tooth structure that may require special attention. As the computed tomography facility was not available at the hospital, intraoperative periapical radiographs with different angles showed the presence of buccal and lingual roots with two distinct canals with #35. The treatment course of the patient was normal, and all the canals were obturated to achieve a three-dimensional hermetic seal. Because of the clinician’s awareness of the probabilities of unusual anatomy, radiographs from different angles were made to assess the root morphology. Further, the authors emphasize the necessity of 3D modeling of the tooth structure if the clinician is expecting any abnormal morphology through radiographic images. Also, training should be employed to assess the tooth 3D images and modeling.

## Conclusions

The clinicians must be able to recognize the existence of unusual and unexpected variations in the root anatomy and canal configuration. To obtain a good clinical outcome and a complete understanding of root canal anatomy and its variations, careful radiographic interpretation associated with clinical inspection is important before initiating RCT. In the present case, exact root morphology and number cannot be apparent with just one 2D radiographic image. Hence, the use of multiple radiographs or computed radiography facility assists in detecting such variations. Conceivably, the clinical success in this case could be accredited to judicious planning, investigation, biomechanical preparation, and obturation of all the canals in both the roots of #35.

## Appendices

Key learning points

1. The detailed clinical and radiographical study is pivotal to detecting deviations from the average or standard root and canal morphology.

2. Though incidences of such aberrations in roots or canals are frequent, the clinician's chairside may encounter an unusual presence.

3. The success of root canal therapy requires proper cleaning and debridement of the infection foci, followed by obtaining an airtight seal.

4. Before initiation of the treatment, knowledge of the root canal system is essential to achieve such objectives.

5. Technological advancements in diagnostic techniques like cone-beam/microcomputed tomography play an essential role in helping dental clinicians to study the root and canal configurations properly.
